# Clinical and radiological features of posterior reversible encephalopathy syndrome in children: a retrospective study

**DOI:** 10.3389/fped.2026.1878823

**Published:** 2026-10-09

**Authors:** Hua Huang, Shujuan Jiang, Huan Xu, Jingmin Zhang, Daogang Qin

**Affiliations:** 1 School of Clinical Medical, Shandong Second Medical University, Weifang, Shandong, China; 2 Department of Pediatrics, Liaocheng People's Hospital, Liaocheng, Shandong, China; 3 Integrated Traditional Chinese and Western Medicine Pediatric Laboratory, Liaocheng People's Hospital, Liaocheng, Shandong, China

**Keywords:** children, emergency management, hypertension, magnetic resonance imaging, posterior reversible encephalopathy syndrome, seizures

## Abstract

**Background:**

Posterior reversible encephalopathy syndrome (PRES) is an uncommon but critical neurological emergency in children. Data on Chinese pediatric cohorts remain limited.

**Objective:**

To analyze the clinical features, imaging findings, and treatment of PRES in children to improve early diagnosis and management.

**Methods:**

A retrospective analysis was conducted on 10 children diagnosed with PRES from March 2016 to January 2025.

**Results:**

The patients included 7 males and 3 females, aged 3–13 years (mean 7.5 years). The main underlying diseases were acute lymphoblastic leukemia (3 cases) and nephrotic syndrome (3 cases). Eight children (80%) had hypertension, 6 (60%) had hypoalbuminemia, and 8 received glucocorticoids or immunosuppressants. The main symptoms were headache, seizures, impaired consciousness, and visual disturbances. Cranial MRI showed symmetrical long T1 and long T2 signals in bilateral posterior cerebral white matter. After comprehensive treatment including antihypertensive, anticonvulsant, and dehydration therapy, all symptoms resolved and lesions disappeared on follow-up imaging without sequelae.

**Conclusion:**

Pediatric PRES is an acute reversible disorder associated with hypertension and immunosuppressive therapy. Early MRI diagnosis and prompt intervention can significantly improve prognosis.

## Introduction

Posterior reversible encephalopathy syndrome (PRES) is a clinico-radiological syndrome characterized by typical manifestations including headache, seizures, altered consciousness, and visual disturbances, with radiological features of reversible vasogenic edema predominantly involving the white matter of the posterior cerebral hemispheres. Its pathogenesis has not been fully elucidated, but is currently considered to be closely associated with endothelial dysfunction, inflammatory responses, and blood-brain barrier (BBB) disruption induced by multiple factors such as hypertension, renal insufficiency, and autoimmune diseases (1). PRES is relatively rare in children, and its exact incidence has not been well established. With early diagnosis and timely treatment, the prognosis is favorable, with complete neurological recovery. However, PRES has an acute onset and rapid progression; delayed diagnosis and treatment may lead to irreversible neurological damage, persistent neurological sequelae, and even life-threatening consequences. In recent years, an increasing number of cases of pediatric PRES have been reported, but systematic clinical analyses focusing on the recognition and systematic management of pediatric PRES remain scarce in China (2). Furthermore, with deeper understanding, novel characteristics have emerged in the etiological spectrum, clinical manifestations, and prognosis of PRES, highlighting an urgent need for updated diagnostic and therapeutic strategies as well as high-quality clinical research (3, 4). Accordingly, this study performed a retrospective analysis of the clinical data from 10 pediatric patients with PRES, summarizing their clinical and radiological features, emergency management measures, and outcomes. The aim is to provide clinical references for pediatric clinicians and improve the capacity for early diagnosis and comprehensive treatment of this condition.

## Materials and methods

### Study subjects

A total of 10 pediatric patients with PRES admitted to the Department of Pediatrics, Liaocheng People's Hospital from March 2016 to January 2025 were enrolled. This study was approved by the Hospital Ethics Committee (Approval No.: 2026026), and written informed consent was obtained from the guardians of all included children.

All patients met the diagnostic criteria for PRES (1, 5), as follows: (1) Presence of known potential underlying predisposing diseases or pathogenic risk factors; (2) Acute or subacute onset, accompanied by typical clinical manifestations such as varying degrees of headache, convulsions, altered mental status, and visual disturbances; (3) Neuroimaging findings (FLAIR/T2WI hyperintensity with corresponding elevated ADC signal) showing vasogenic edema predominantly involving the posterior white matter of the bilateral cerebral hemispheres; (4) Clinical symptoms and neuroimaging changes were reversible after active and rational treatment; (5) Exclusion of other possible white matter lesions. Inclusion criteria: Meeting the above-mentioned diagnostic criteria for PRES. Exclusion criteria: A history of pre-existing white matter lesions, hereditary encephalopathy, or definite central nervous system infection.

### Data collection

(1) Basic data included name, sex, age, underlying diseases, usage of immunosuppressive agents, disease course, and therapeutic measures; (2) Relevant clinical manifestations at the onset of PRES, including onset pattern, neurological symptoms and signs, blood pressure, etc.; (3) Main auxiliary examinations, including complete blood count, blood biochemistry, electroencephalogram, cranial MRI and other imaging data; (4) Treatment and outcome: therapeutic methods, time to improvement, presence or absence of recurrence, and follow-up results.

### Study methods

A retrospective analysis was performed. The basic data, clinical manifestations, cranial imaging characteristics, treatment and follow-up data of the children were collected and organized, and analyzed and discussed in combination with relevant literature reports.

## Results

### General data

Among the 10 children, 7 were male and 3 were female. The age at onset ranged from 3 to 13 years, with a mean age of 7.5 years. Primary diseases included acute lymphoblastic leukemia in 3 cases, nephrotic syndrome in 3 cases, and lupus nephritis, hemophagocytic syndrome, hypertension, and infection-associated encephalopathy in 1 case each. Eight children had received glucocorticoids or combined immunosuppressive therapy before the onset of PRES, among whom 5 were treated with glucocorticoids combined with immunosuppressive agents, and 3 were treated with glucocorticoids alone.

### Clinical manifestations

All children presented with acute onset. The main clinical manifestations included: convulsions in 8 cases (80%), among whom 5 had recurrent seizures or status epilepticus; headache (with or without vomiting) in 7 cases (70%); disturbance of consciousness (drowsiness, lethargy, coma) in 7 cases (70%); visual disturbance in 3 cases (30%). Eight cases (80%) exhibited significantly elevated blood pressure at onset, exceeding the 95th percentile for age- and sex-matched children. Detailed clinical data of the patients are shown in Tables 1, 2.

**Table 1:** Demographic characteristics, underlying diseases, blood pressure, and serum protein levels of 10 pediatric patients with PRES.

Case	Sex	Age (y)	Underlying disease	Disease course	Previous drug therapy	Bp at onset (mmHg) (mmHg)	Serum protein (g/L) (g/L)
1	Female	5	ALL	6 months	VDLD delayed chemotherapy	129/85	34
2	Female	13	ALL	17 months	Glucocorticoid + methotrexate + mercaptopurine for 7 months	122/71	39
3	Male	6	ALL	1 month	VDLD induction therapy	140/90	25
4	Male	7	NS	1 year	Glucocorticoid (1 year) + glucocorticoid pulse therapy for 12 days+oral cyclosporine for 1 month	180/120	29
5	Male	12	NS	3 months	Glucocorticoid + multiple cyclophosphamide pulse therapies, oral tacrolimus for 16 days	161/122	17
6	Male	9	NS	3 months	Oral glucocorticoid for 5 days	195/130	15
7	Male	3	Hemophagocytic syndrome	14 days	Intravenous etoposide for 5 doses + glucocorticoid for 1 month + oral cyclosporine for 7 days	150/110	37.2
8	Male	3	Viral encephalopathy	1 day	No treatment	87/45	42
9	Male	5	Hypertension	8 days	No treatment	140/100	47
10	Female	12	Lupus nephritis	2 months	Oral glucocorticoid + tacrolimus for 2 months	150/105	30

ALL, Acute Lymphoblastic Leukemia; NS, Nephrotic Syndrome; VDLD, vincristine, daunorubicin, L-asparaginase, dexamethasone.

**Table 2:** Neurological symptoms, laboratory findings, imaging, treatment, and outcomes of 10 pediatric patients with PRES.

Case	Neurological symptoms	Seizure type/Status epilepticus	BUN (mmol/L)	Cr (μmol/L)	Na (mmol/L)	MRI	Treatment	Prognosis
1	Headache, vomiting	None	7.8	22	128	Bilateral frontal lobes, trigones of lateral ventricles	Dehydration, antihypertensive therapy	Recovered
2	Headache, nausea	None	4.48	23.5	135.3	Bilateral frontal lobes	Dehydration	Recovered
3	Headache, vomiting, seizures, altered consciousness	Focal/No	3.4	18	113.1	Bilateral parietal and occipital lobes	Dehydration, antihypertensive therapy, anticonvulsant therapy	Recovered
4	Headache, dizziness, vomiting, ocular discomfort, seizures, coma	Generalized/Yes	6.6	24	131	Bilateral frontal, parietal, occipital, temporal lobes and thalamus	Dehydration, antihypertensive therapy, anticonvulsant therapy, discontinuation of cyclosporine	Recovered
5	Headache, dizziness, vomiting, blurred vision, seizures	Generalized/Yes	21.2	99	134.9	Bilateral frontal, parietal, and occipital lobes	Dehydration, antihypertensive therapy, anticonvulsant therapy, discontinuation of tacrolimus	Recovered
6	Headache, dizziness, seizures, lethargy	Generalized/Yes	7.08	100.7	136.4	Bilateral parietal lobes	Dehydration, antihypertensive therapy	Recovered
7	Headache, seizures, altered consciousness	Generalized/Yes	6.93	13.3	131.9	Bilateral frontal, parietal, and occipital lobes, centrum semiovale	Dehydration, antihypertensive therapy, anticonvulsant therapy, discontinuation of cyclosporine	Recovered
8	Seizures, altered consciousness	Generalized/Yes	2.62	15.9	133.9	Bilateral temporal lobes, corpus callosum, centrum semiovale	Dehydration, anticonvulsant therapy	Recovered
9	Blurred vision, seizures, altered consciousness	Generalized/Yes	1.63	29.2	124.6	Bilateral frontal, parietal, and occipital lobes	Dehydration, antihypertensive therapy, anticonvulsant therapy	Recovered
10	Vomiting, seizures, altered consciousness	Generalized/Yes	10.41	51.6	136.4	Bilateral frontal, parietal, occipital, and temporal lobes	Dehydration, antihypertensive therapy, anticonvulsant therapy, discontinuation of tacrolimus	Recovered

BUN, Blood Urea Nitrogen; Cr, Creatinine; Na = Sodium.

### Imaging findings

All children underwent cranial MRI within 48 h after the onset of neurological symptoms. Imaging revealed that lesions were mostly symmetric and multifocal, presenting as patchy or large confluent areas. All patients showed symmetric long T1 and long T2 signals in the posterior white matter of bilateral cerebral hemispheres, mainly involving the parieto-occipital and frontal lobes. Some patients also had lesions in the temporal lobe, centrum semiovale, corpus callosum, bilateral thalamus, and trigone of the lateral ventricle. The imaging data of Case 5 and Case 9 are shown in Figures 1–4, respectively.

**Figure 1:**
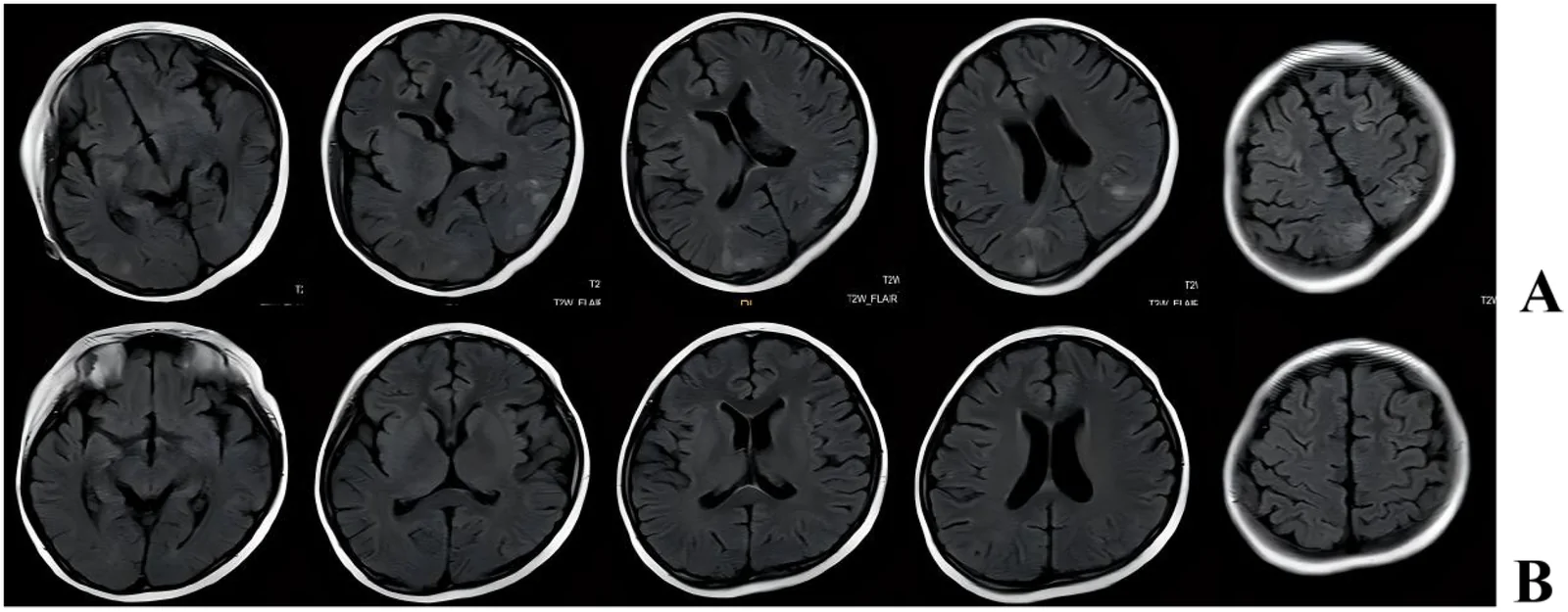
Cranial MRI manifestations (FLAIR sequence) of case 5 before and after treatment. **(A)**: Cranial MRI at onset showed multiple approximately symmetric hyperintense signals on FLAIR sequence in bilateral frontal, parietal and occipital lobes. **(B)**: MRI after 20 days of treatment showed obvious absorption of the lesions compared with the previous images.

**Figure 2:**
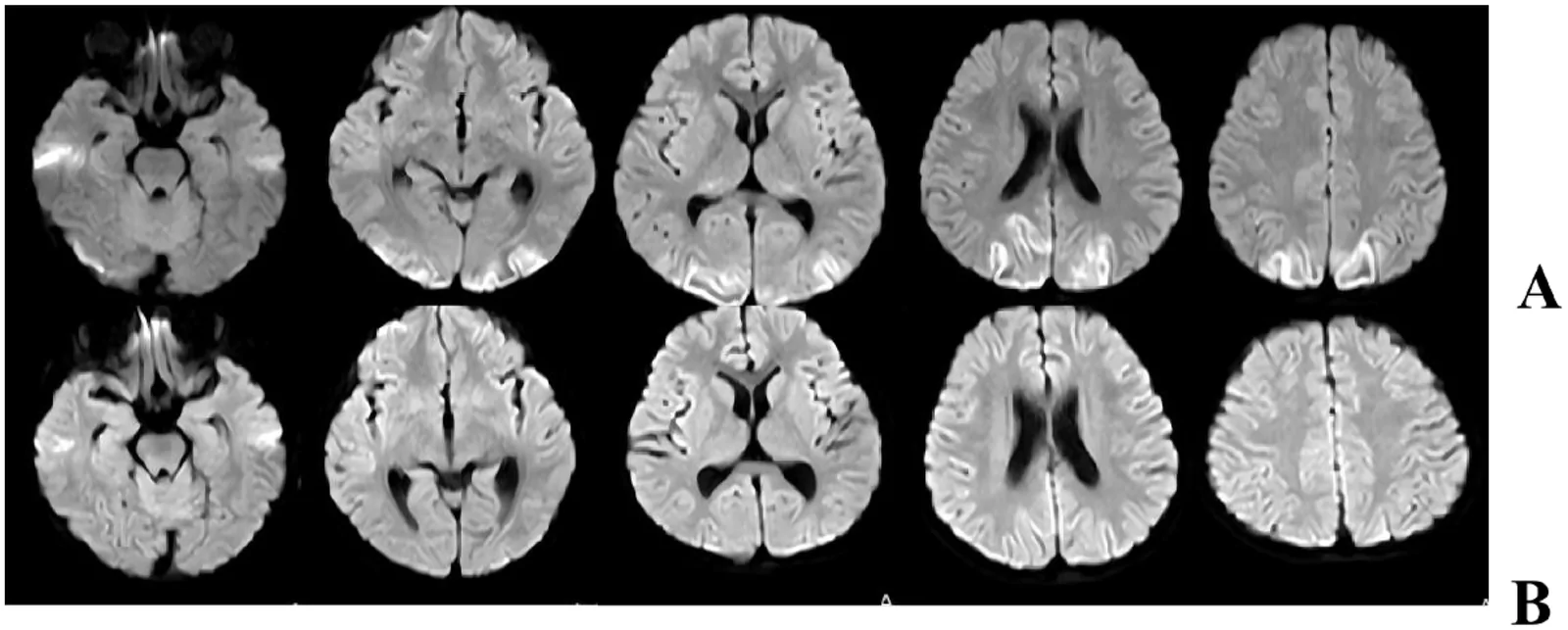
Cranial MRI manifestations (DWI sequence) of case 5 before and after treatment. **(A)**: Cranial MRI at onset showed multiple approximately symmetric hyperintense signals on DWI sequence in bilateral frontal, parietal and occipital lobes. **(B)**: MRI after 20 days of treatment showed obvious absorption of the lesions compared with the previous images.

**Figure 3:**
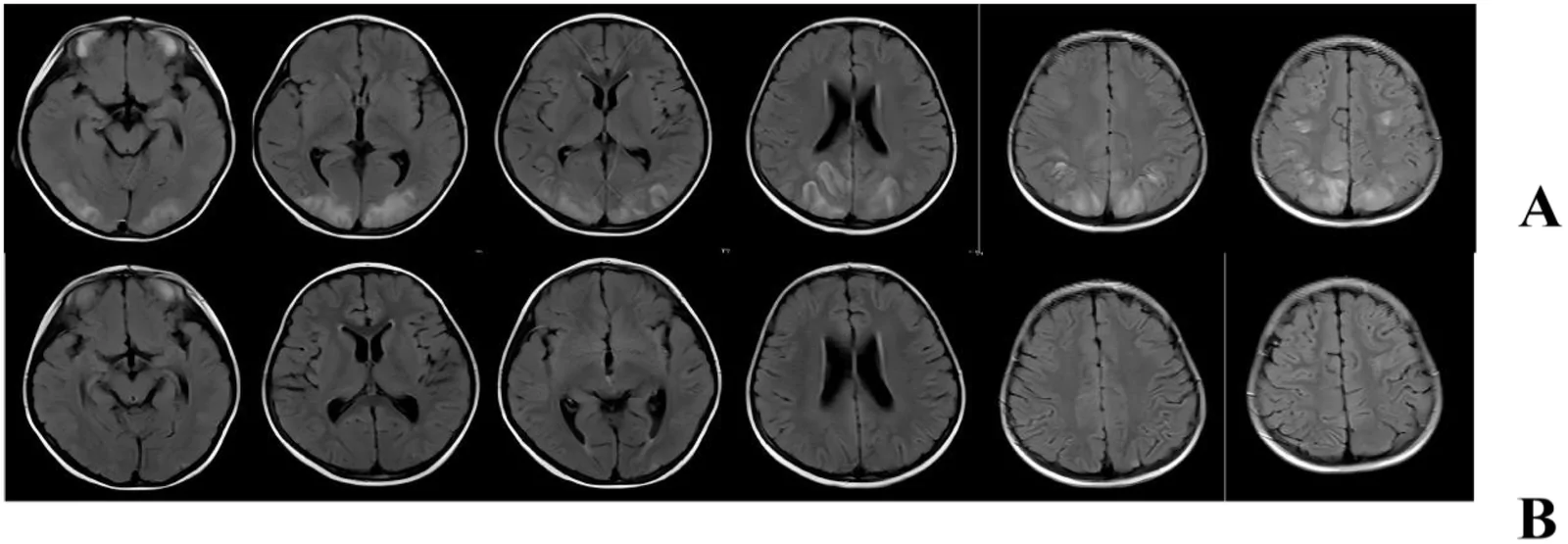
Cranial MRI manifestations (FLAIR sequence) of case 9 before and after treatment. **(A)**: Cranial MRI at onset showed symmetric patchy slightly hyperintense signals on FLAIR sequence in the cortical regions of bilateral frontal, parietal and occipital lobes. **(B)**: MRI after 20 days of treatment showed obvious absorption of the lesions compared with the previous images.

**Figure 4:**
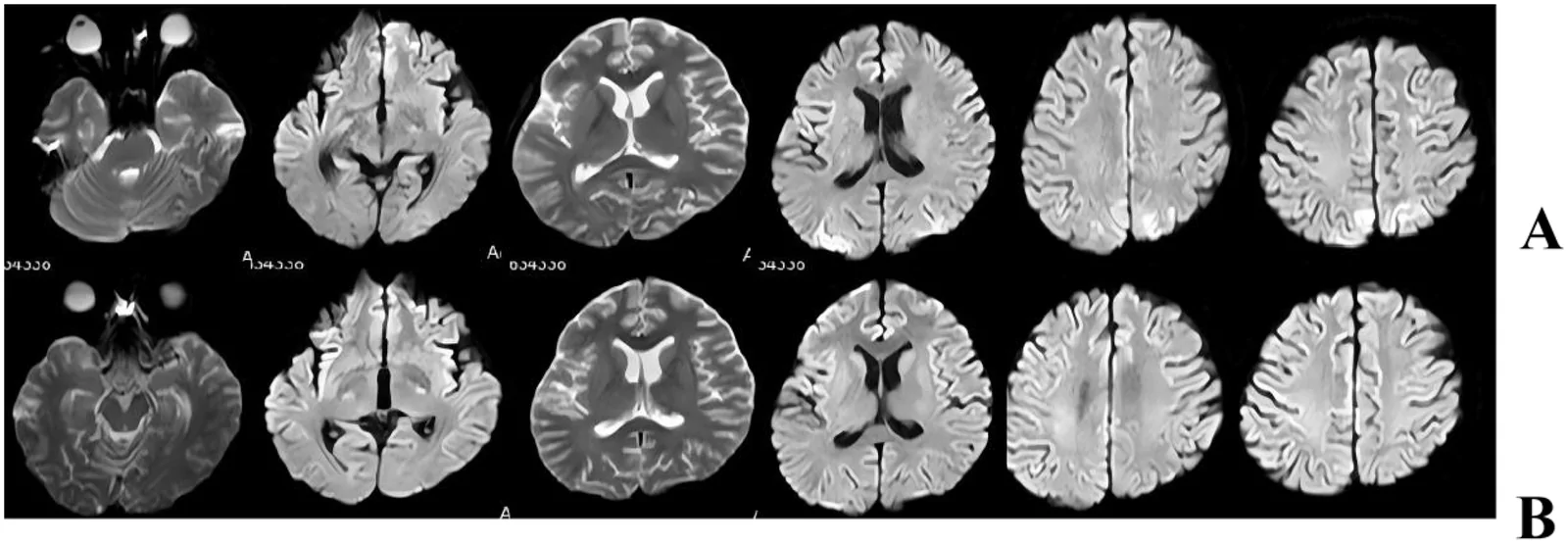
Cranial MRI manifestations (DWI sequence) of case 9 before and after treatment. **(A)**: Cranial MRI at onset showed symmetric patchy slightly hyperintense signals on DWI sequence in the cortical regions of bilateral frontal, parietal and occipital lobes. **(B)**: MRI after 20 days of treatment showed obvious absorption of the lesions compared with the previous images.

### Laboratory examinations

Hypoalbuminemia and electrolyte disturbances (e.g., hyponatremia) were present in 6 patients. Renal impairment, manifested as abnormally elevated urinary protein, creatinine, and urea nitrogen, was observed in 4 cases. No abnormalities were found in cerebrospinal fluid examination in any patient.

### Treatment and prognosis

All patients received aggressive dehydration, antihypertensive therapy, treatment of primary diseases, and symptomatic management. Antihypertensive agents included calcium channel blockers, diuretics, and angiotensin receptor antagonists. Patients with convulsions received diazepam (0.3–0.5 mg/kg per dose, intravenous injection) or midazolam (0.1–0.2 mg/kg per dose, intravenous injection; continuous intravenous infusion at 1–5*μ*g/kg·min for status epilepticus) for emergency seizure control. One patient was treated with levetiracetam and oxcarbazepine for seizure control. After active treatment, with a mean follow-up duration of 12 months, all patients achieved significant symptomatic relief. Follow-up neuroimaging demonstrated resolution of lesions, with no neurological sequelae.

## Discussion

The name PRES originates from its typical neuroimaging manifestation, namely symmetric edema in the posterior white matter of the brain. This syndrome was first systematically described by Hinchey et al. (6) in 1996. Its clinical features include headache, confusion or decreased level of consciousness, visual disturbances, and seizures, with neuroimaging demonstrating posterior cerebral white matter edema. Notably, PRES lesions are not confined to the posterior cerebrum and may also involve the frontal lobe, temporal lobe, basal ganglia, and other regions (6, 7).

The pathogenesis of PRES is complex, and current studies suggest that multiple factors are involved (8). Hypertension is one of the key factors. The cerebral vascular autoregulatory capacity is immature in children, and a rapid elevation in blood pressure can easily lead to hyperperfusion, disruption of the BBB, and subsequent vasogenic edema (9, 10). In the present study, 80% of children presented with hypertension, which is consistent with these findings. Importantly, PRES is not exclusively a hypertensive disorder. Approximately 15%–30% of pediatric cases occur in children with normal baseline blood pressure, triggered instead by immunosuppressive agents, severe infections, or autoimmune inflammatory conditions (11). Case 8 in our series, who presented with infection-associated encephalopathy and sustained normotension throughout the acute phase, exemplifies this normotensive form. Furthermore, the rate of blood pressure elevation may be more clinically significant than the absolute value. A rapid surge from a normal or even low baseline can overwhelm the immature cerebral autoregulatory capacity in children more readily than sustained moderate hypertension, leading to breakthrough hyperperfusion and BBB disruption (12, 13). This underscores the importance of obtaining a thorough baseline blood pressure history and recent hemodynamic trajectory—rather than relying solely on a single emergency department measurement—when assessing a child for possible PRES. Renal insufficiency and internal environmental disturbances are closely associated with the development of PRES. When renal function is impaired, accumulated metabolic wastes and toxins in the body may interfere with cerebral vessels and increase the risk of PRES (14). Three children in this group had elevated serum creatinine and urea nitrogen, supporting this view. Autoimmune diseases can affect cerebral vessels through immune-mediated inflammatory responses, impairing vascular structure and function, and thereby leading to PRES (15). The primary diseases in this cohort included lupus nephritis, hemophagocytic syndrome, and nephrotic syndrome. In addition, infection and immunosuppressive agents may also be important triggers. Three children in this group had a history of infection before onset, and most had received glucocorticoids or immunosuppressive therapy. In summary, the present study supports the concept that multiple factors contribute to the pathogenesis of PRES (2). Further studies are still needed to fully elucidate the pathogenesis of PRES and provide more sufficient evidence for its prevention and treatment.

In our cohort, the clinical manifestations were characterized by convulsions (80%), headache (70%), altered consciousness (70%), and visual disturbances (30%). While these overall proportions are broadly in line with the literature (16–18), the rank order of symptoms in our series is not entirely consistent with some previous reports. Specifically, in our patients, headache (70%) and altered consciousness (70%) occurred with equal frequency and both followed seizures, whereas several prior pediatric PRES series have reported that encephalopathic features (rather than headache) are the second most common presentation after seizures, with headache and visual disturbances being less frequent. This variation may reflect the inherent limitations of our retrospective, single-center design, including caregiver recall bias and differences in the timing of presentation to our emergency department. Notably, subtle encephalopathic features such as lethargy or confusion may have been underrecognized by parents at the initial stage, whereas headache could be more readily verbalized by older children. Nonetheless, seizures consistently emerge as the predominant and most alarming manifestation of pediatric PRES across the literature, a finding that our data corroborate (11). The occurrence of these symptoms may be related to edema in the posterior white matter of the brain, especially the parieto-occipital lobe, involving the visual pathways, cortical and subcortical structures, leading to impaired neural conduction (19, 20). As PRES lacks specific clinical manifestations, it can be easily confused with other diseases such as intracranial infection and metabolic encephalopathy (9, 14). Therefore, PRES should be highly suspected in children with acute onset, a history of hypertension or immunosuppressant use, and neurological symptoms, especially in those with underlying diseases including nephrotic syndrome, leukemia, and lupus nephritis. Cerebrospinal fluid in PRES patients is usually normal with no evidence of infection. Neuroimaging features include symmetric involvement of the posterior white matter in the parieto-occipital lobes with vasogenic edema, mostly no marked hyperintensity on DWI, and hyperintensity on ADC. Lesions can resolve completely within a short period after active treatment (21, 22). In contrast, demyelinating diseases usually present with subacute onset and asymmetric lesions, which may involve deep white matter, brainstem, and spinal cord. DWI may show hyperintensity with isointensity or hypointensity on ADC. Recovery is slow after steroid therapy, with a tendency to relapse or leave permanent lesions (22). The clinical and imaging evolution in the present cases was consistent with the typical features of PRES, and no demyelinating changes were observed.

MRI is crucial for the diagnosis of pediatric PRES, with typical manifestations including symmetric long T1, long T2, and FLAIR hyperintensities in the posterior white matter of the bilateral cerebral hemispheres. DWI is essential for differential diagnosis. Since PRES is centered on vasogenic edema, DWI mostly shows isointensity or slight hyperintensity, which can be clearly distinguished from cytotoxic edema in cerebral infarction (marked DWI hyperintensity) (2, 23). In the present study, MRI findings of all children were consistent with these characteristics. The characteristic parieto-occipital predominance of PRES lesions is thought to reflect the relatively sparse sympathetic innervation of the posterior cerebral circulation. Compared with the anterior circulation, the posterior arterioles have less capacity for autoregulatory vasoconstriction during acute hypertension, rendering them preferentially vulnerable to hyperperfusion injury (24). In our cohort, nearly all lesions were centered in watershed (border-zone) territories—the junctional zones between major cerebral arterial supplies—which are particularly susceptible to perfusion pressure fluctuations. This pattern is well illustrated by the bilateral symmetric FLAIR hyperintensities in Figures 1–4. Beyond the classic posterior pattern, we observed two additional imaging phenotypes in our more severely affected patients: the diffuse holohemispheric watershed pattern (Cases 4, 5, 7, 9, and 10) and the deep central gray matter pattern (thalamic involvement in Case 4). The holohemispheric pattern reflects widespread microcirculatory failure and more extensive BBB disruption, while central gray matter involvement—including the basal ganglia, thalami, cerebellum, and brainstem—has been associated with advanced PRES and a higher risk of transient neurological deterioration. These extensive distributions likely represent progressive endothelial injury resulting from sustained or severe hyperperfusion (25–27). Lesions mainly involved the parieto-occipital and frontal lobes, and some cases also involved the temporal lobe, corpus callosum, and other regions. Combined with acute onset, typical clinical symptoms, underlying diseases, and normal cerebrospinal fluid findings, an accurate diagnostic framework can be established to avoid misdiagnosis.

There is no specific treatment for PRES, and comprehensive management is emphasized, with the core being early recognition and a multimodal approach. Stable blood pressure control is the cornerstone of treatment. The goal is not merely to achieve numerical targets, but to restore cerebral vascular autoregulation and interrupt the vicious cycle of BBB damage. The cerebral vascular autoregulatory mechanism in children is immature, with a lower upper limit of regulation than in adults, rendering them more sensitive to blood pressure fluctuations (28). According to the Chinese Reference Standards for Blood Pressure in Children (28), the 95th percentile ranges from 115 to 135/75–85 mmHg. Most children had blood pressure significantly exceeding age-appropriate thresholds with acute neurological symptoms, indicating decompensated autoregulation that required active intervention. An excessively rapid reduction in blood pressure should be avoided to prevent insufficient cerebral perfusion, while an overly slow reduction may aggravate edema (29). In this study, calcium channel blockers were the first-line agents (amlodipine 0.1–0.3 mg/kg/d, orally or via nasogastric tube in 1–2 divided doses). Angiotensin receptor antagonists (valsartan 0.5–1 mg/kg/d) were added in children with markedly elevated blood pressure (>160 mmHg) or status epilepticus. Potent vasodilators such as sodium nitroprusside were avoided (2, 28). Continuous blood pressure monitoring was performed during treatment to maintain blood pressure at the 90th–95th percentile for age or a 20%–25% reduction from baseline, which ensured adequate cerebral perfusion while avoiding hyperperfusion injury (28, 29). Blood pressure reached target levels within 24–48 h in all children, with no hypotensive complications.

Although hypertensive hyperperfusion is a primary driver of PRES, emerging evidence points to an additional intracellular metabolic cascade that amplifies vascular injury. Systemic stressors—including those from underlying diseases, immunosuppressants, and infections—can induce endoplasmic reticulum (ER) stress and mitochondrial dysfunction, leading to unregulated Ca^2^⁺ release from the ER into the cytoplasm (30). This intracellular calcium overload triggers endothelial cell apoptosis, degradation of the vascular basement membrane, and vasoparalysis of vascular smooth muscle, collectively compromising BBB integrity and promoting vasogenic edema (31). This calcium-dependent injury pathway provides a mechanistic rationale for the first-line use of calcium channel blockers (CCBs) in pediatric PRES: CCBs not only lower systemic blood pressure but may also attenuate intracellular calcium overload in cerebrovascular endothelial and smooth muscle cells, thereby preserving microvascular structural integrity (32). This ER-Ca^2^⁺-mitochondrial cascade may also help explain rare cases of PRES in which clinical recovery is delayed despite prompt blood pressure normalization, because residual intracellular calcium overload and sustained ER stress can continue to compromise endothelial function for hours to days after systemic hypertension has been controlled (30, 33). In our cohort, all patients received early CCB-based combination therapy, which may have concurrently addressed both hemodynamic and calcium-mediated injury—potentially contributing to the complete resolution we observed. However, among patients with more advanced activation of this cascade, blood pressure reduction alone may be insufficient to reverse established endothelial damage, which could account for occasional reports of persistent neurological sequelae despite timely antihypertensive treatment (34).

Seizure control is equally critical. The incidence of seizures in children with PRES is as high as 70%–80% (17). The underlying mechanisms involve abnormal cortical excitability due to hyperperfusion, edema extending to the visual cortex and adjacent association areas, BBB disruption, and electrolyte disturbances (2). Seizures and cerebral hyperperfusion form a vicious cycle, making early and rapid termination crucial (1). In the present study, 8 children with seizures received intravenous diazepam (0.3–0.5 mg/kg, maximum 10 mg) or intravenous midazolam (0.1–0.2 mg/kg) as first-line therapy. Among them, 5 cases with status epilepticus were treated with continuous intravenous midazolam infusion at 1–5 μg/kg·min. One patient with frequent recurrent seizures received combined oral levetiracetam (10–20 mg/kg) and oxcarbazepine (10 mg/kg). Concomitant aggressive antihypertensive and dehydrant therapy was administered to eliminate the precipitating factors of seizures. Seizures were completely controlled within 24–48 h in all children, with no requirement for long-term antiepileptic treatment, reflecting the reversible nature of PRES-associated seizures.

Adjustment of immunosuppressive agents represents an important component of etiological treatment. Cyclosporine A, tacrolimus, and similar agents can damage vascular endothelial cells (35), leading to disturbed vasomotor function and increased vascular permeability (4). Their nephrotoxicity results in water and sodium retention, which, together with the synergistic effect of glucocorticoids, can cause a rapid elevation in blood pressure (2, 10). Among the 5 children treated with these agents in the present study, 4 exhibited markedly elevated blood pressure accompanied by renal dysfunction, consistent with the drug-vascular-blood pressure interactive injury pattern. Therefore, in children with a definite association with such agents, immediate discontinuation and substitution with less nephrotoxic alternatives are recommended (4, 36, 37). For those receiving glucocorticoid monotherapy, gradual tapering is required to avoid risks associated with abrupt withdrawal. The 4 children in this cohort who underwent timely discontinuation of cyclosporine A or tacrolimus showed a favorable prognosis.

Meanwhile, active treatment of the primary disease is fundamental to preventing recurrence (38, 39). In the present study, 3 children with PRES induced by nephrotic syndrome achieved symptomatic relief within several days after discontinuation of nephrotoxic immunosuppressants and aggressive control of blood pressure and seizures, and immunosuppressive therapy for the primary disease was continued without further recurrence. Literature also supports that children with systemic lupus erythematosus complicated by PRES can achieve complete remission with standard glucocorticoid therapy (40).

Comprehensive supportive care, including symptomatic management such as correction of internal environmental disturbances and reduction of intracranial pressure, is also essential (41). In the present study, 6 children with concurrent hyponatremia showed symptomatic improvement after correction of electrolyte disorders. For those with obvious signs of increased intracranial pressure, intravenous infusion of mannitol (0.5–1 g/kg, repeatable every 6–8 h) or furosemide (1 mg/kg) effectively relieved symptoms by reducing intracranial pressure. All children in this cohort achieved significant symptomatic relief after the above-mentioned comprehensive treatment. With a mean follow-up duration of 12 months, neuroimaging showed resolution of lesions, and no neurological sequelae were observed. This confirms the importance of early recognition and systematic intervention in improving prognosis.

## Limitations

This study has several limitations. First, the limited sample size (*n* = 10) constrains the generalizability of our observed symptom proportions. The high prevalence of seizures (80%) and headache (70%) in this cohort may not reflect the full spectrum of early PRES presentations across diverse pediatric populations. Interstudy heterogeneity in the rank order of clinical manifestations is widely documented in published pediatric PRES case series, highlighting the need for larger, multicenter studies to define more precise epidemiological patterns. Second, as a retrospective study, there may be selection bias and incomplete data recording. Third, the lack of a control group precludes causal inference regarding risk factors. Fourth, long-term follow-up beyond 12 months was not available for all patients. Future multicenter prospective studies with larger cohorts are needed to validate our observations.

## Patient perspective

The guardians of the included children provided written informed consent and reported that all neurological symptoms (seizures, headache, and altered consciousness) resolved completely within 2–4 weeks after treatment. No child experienced recurrence or long-term functional impairment during the 12-month follow-up period.

## Conclusions

Pediatric PRES is a reversible but neurologically emergent condition that requires urgent management. Its occurrence is closely associated with multiple factors, including hypertension and immunosuppressive therapy. Successful treatment relies on high clinical suspicion, timely cranial MRI examination, and a comprehensive management strategy centered on blood pressure and seizure control, while also addressing the primary disease and maintaining internal environmental homeostasis. For children presenting with acute seizures, headache, altered consciousness, or visual disturbances, especially those with a history of hypertension or immunosuppressant use, PRES should be prioritized in the differential diagnosis and prompt MRI obtained. Improving pediatricians’ awareness of this disorder and establishing a rapid diagnosis and treatment pathway is of great importance for improving the prognosis of affected children.

## Data Availability

The original contributions presented in the study are included in the article/Supplementary Material, further inquiries can be directed to the corresponding authors.
